# Self–handicapping among nursing students: an interventional study

**DOI:** 10.1186/s12909-018-1441-6

**Published:** 2019-04-01

**Authors:** Ladan Zarshenas, Leila Ashrafean Jahromi, Mohsen Faseleh Jahromi, Marieh Dehghan Manshadi

**Affiliations:** 1 Community-based Psychiatric Care Research Center, School of Nursing and Midwifery, Shiraz University of Medical Sciences, Shiraz, Iran; 2 Department of Nursing, School of Nursing and Midwifery, Student Research Committee, Shiraz University of Medical Sciences, Shiraz, Iran; 3 Department of Nursing, School of Nursing and Paramedical Sciences, Jahrom University of Medical Sciences, P.O.Box 7154341508, Jahrom, Iran; 4 Department of Humanistic Sciences, Faculty of Human Sciences, Islamic Azad University of Yazd, Yazd, Iran

**Keywords:** Problem solving, Nursing, Students

## Abstract

**Background:**

Self-handicapping is an effective defense strategy in an individual’s behavior that leads to weak performance in different situations like education. This study aimed to investigate how training problem solving skills affected the rate of self-handicapping among nursing students.

**Methods:**

This interventional study was done in Jahrom, Fars province, Iran during 2016–2017. Totally, 90 nursing students were selected among those admitted from 2013 to 2016 using stratified sampling. Then, the students were randomly divided into a control and an intervention group each including 45 participants. Teaching problem solving skills to the intervention group was completed over six sessions each lasting for two hours. The students’ rate of self-handicapping was evaluated based on the scores obtained in Jones and Rodwalt’s self-management questionnaire before and after the intervention (immediately and one month later). The data were entered into the SPSS statistical software, version 16 and were analyzed using descriptive and inferential statistics, including t-test, chi-square, and repeated measures ANOVA. The significance level was set at 0.05.

**Results:**

The findings revealed a significant difference in the intervention group’s self-handicapping scores before and after the intervention (*p* < 0.001). However, no significant change was observed in this regard in the control group (*p* = 0.575). The results indicated no significant differences between the intervention and control groups concerning the mean score of self-handicapping immediately after the intervention (*p* = 0.761). However, a significant difference was detected between the two groups in this regard one month after the intervention (*p* = 0.014).

**Conclusion:**

Teaching problem solving skills influenced the students’ beliefs and performances positively and led to a decrease in their self-handicapping. Thus, teaching cognitive-behavioral approaches is recommended to be considered among the ten life skills used in curricular design for medical students, including nurses.

**Trial registration:**

IRCT 2017011231895 N.Data registered: October 30, 2016.

## Background

Today, psychologists play an important role in investigating and improving psychological behaviors and problems [1]. Meanwhile, one of the most important applied situations in psychology is the cases dealing with education [1]. On the other hand, one of the purposes of education is gaining educational achievements beside maintaining and improving the psychological health of school and university students. Hence, identifying problems and variables related to these factors are of high significance [2, 3]. Fear from failure is among such problems. Researchers believe that people who are afraid of failure choose situations in which they can attribute success to their own ability and failure to external factors in order to keep their self-esteem [4]. One of the less-known strategies to justify failure is self-handicapping [5]. Self-handicapping is a defense strategy that was first introduced by Berglas and Jones (1978). These researchers defined self-handicapping as creating or claiming an obstacle for successful performance of tasks [6]. Therefore, people who create or claim the existence of obstacles try to show that there is no relation between a probable failure and their capabilities [7]. In other words, self-handicapping involves any activity or performance that one uses for increasing the opportunity to provide an excuse for one’s failure and to attribute failure to external and success to internal factors [8]. Hence, this strategy impels individuals toward a conscious decision-making whose result can distort others’ beliefs [9]. Making an effort acts as a double-edged sword for these people. On one hand, their efforts can lead to success. On the other hand, failure in their efforts will negatively affect their self-efficacy [10].

Evidence has indicated that using self-handicapping strategies would cost a lot, including lack of assurance in one’s own capabilities, losing hope to repeat one’s previous successes, being impatient, using drugs, magnifying illnesses and pains, low self-confidence, low psychological health level, and low life satisfaction [11, 12].

Researchers have shown an increase in self-handicapping when it is to be evaluated [13]. In this respect, some studies reported the mutual effects of self-handicapping and educational performance [14, 15]. Some other studies have also revealed that self-esteem was in danger in this realm as in other settings. Thus, people might use self-handicapping strategies in order to manage this threat [12]. In the same vein, some researchers have shown that self-handicappers were educationally weak [16, 17].

The opposite of self-handicapping is self-efficacy, which is the most important factor in displaying any behavior [18]. Cognition plays a vital role in self-handicapping and self-efficacy, such a way that it influences individuals’ understanding of situations and behaviors. Cognitive approaches refer to the rules and principles of processing information when confronting various stimuli. It is believed that individuals’ cognitive processes and beliefs determine their conditions or feelings in different situations [19]. Therefore, approaches like problem solving, which are based on cognitive methods, are expected to play a basic role in students’ self-efficacy and self-handicapping [20]. Teaching problem solving strategy that started since the late 1960s was a part of the cognitive-behavioral movement [21], insisting on a cognitive-behavioral process that provided potential solutions to a difficult situation and, consequently, increased the possibility of choosing the most effective solution [22]. In fact, teaching problem solving can be defined as the process of helping a person develop one’s learning and consequently increase the probability of effective confrontation in a vast range of situations [23]. Problem solving is a type of confrontation concentrating on a specific problem that is used by individuals for decision-making. After people define a problem and different solutions, they will choose one of the solutions [24]. Problem solving is the basis of nursing processes [25] because nursing students face many challenges in medical environments. Considering the probability of nurses’ lack of adaptation to such situations, development of this issue is of particular importance among nurses [26].

Based on what was mentioned above, using self-handicapping strategies is one of the important and effective factors in students’ psychological health that has important consequences, such as weak educational performance and lack of efforts. Considering the effective role of cognitive approaches in the way students face difficult situations, the present study aims to investigate the effect of teaching problem solving skills on the rate of self-handicapping among nursing students.

## Methods

### Study design

This two-group interventional study was conducted in the form of pretest and posttest. The research community included all senior nursing students studying in Jahrom University of Medical Sciences during 2016–2017.

### Sampling

**Fig. 1:**
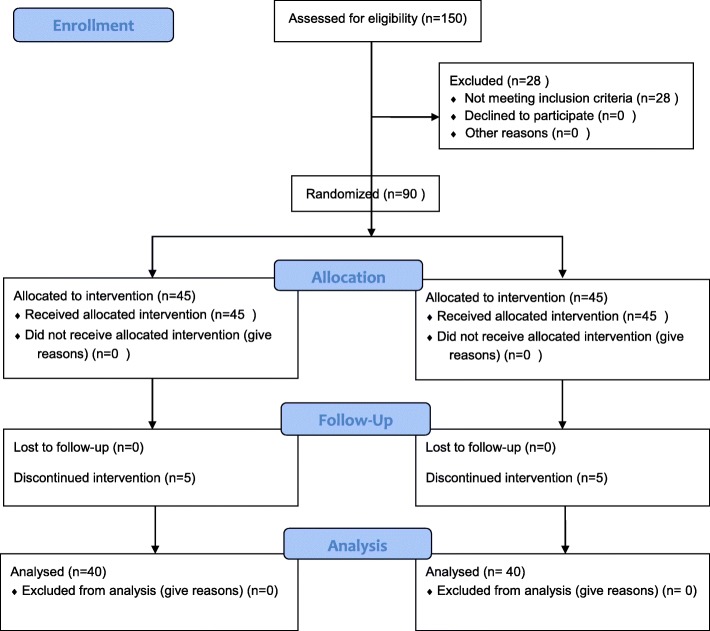
Flow chart of the study design

Considering α = 0.05, confidence level of 90%, and probability of 5% loss and using PASS11 NCSS software, a 90-subject sample size was determined for this study (45 participants in each group). The self-handicapping test was given to all nursing students (*n* = 150) registered from 2013 to 2016. Accordingly, 122 students whose self-handicapping grades were higher than 50 were identified. Then, based on the number of self-handicappers in each class, 90 subjects were selected through proportional stratified randomization and were randomly assigned to the intervention and control groups using the table of random numbers. Due to different number of self-handicappers in each education level, first the number of participants from each level of education was determined through stratified sampling method. Then, simple random sampling was done by drawing from the numbers assigned to each person at each education level. These subjects were randomly assigned to an intervention and a control group each containing 45 participants. (Fig. 1). The inclusion criteria of the study were being an active student in Jahrom University of Medical Sciences, getting a self-handicapping grade higher than 50, and being willing to participate in the study. The exclusion criteria were not attending the educational classes and not being able to pass the problem solving program.

### Instruments

The study data were collected using a demographic characteristics form and Jones and Rodwalt’s self-handicapping scale. The demographic characteristics form included items, such as age, gender, residence, marital status, the current semester’s average score, the mean of the previous semesters’ average scores, parents’ education levels, and experienced tensions during the past six months.

Jones and Rodwalt’s self-handicapping scale (1982) consisted of 23 questions for evaluating individuals’ tendency towards self-handicapping. The items were responded through a Likert scale ranging from completely agree (0) to completely disagree [5]. The items were divided into three subscales, namely ill temper, lack of endeavor, and excusing. The sum of scores of items 4, 7, 8, 9, 13, 15, 19, 20, and 23 indicated the ill temper subscale, sum of the reverse scores of items 3, 5, 6, 10, 17, 21, and 22 represented the lack of endeavor subscale, and sum of the scores of items 1, 2, 11, 12, 14, 16, and 18 indicated the excusing subscale. Accordingly, scores 0–50, 51–100, and > 101 represented low, medium, and high self-handicapping, respectively [27].

### Validity and reliability

Jones and Rodwalt’s self-handicapping scale was translated and its psychometric properties were assessed by Heidari et al. (2009). In order to determine the construct validity of the instrument, 23 out of the 25 items were put on three factors; i.e., “ill temper”, “endeavor”, and “excusing”, and two items were omitted because they did not load on any of the factors. The sum of scores for ill temper and excusing factors and the reverse scores of endeavor factor represented general self-handicapping. Applying the self-handicapping scale with a 15-day interval showed highly significant correlations between the factors, subscales, and the whole self-handicapping scale in the two applications, which ranged from 0.47 for the endeavor factor to 0.86 for the total scale. Additionally, investigating the reliability of the scale using internal consistency revealed alpha to be 0.6 for endeavor and excusing factors and 0.77 for the total self-handicapping scale, representing an acceptable internal consistency. The reliability coefficient was also 0.84, indicating the reliability of the scale [27].

### Procedure

After getting permission from the University, the research procedures were explained to the participants by one of the trained researchers in the study. Then, the intervention group participants were enrolled into a training course for problem solving based on Dezorilla and Goldfried model. This model had six stages that were held in six two-hour sessions within six weeks. Necessary trainings in each session were offered separately using lecture method, group discussion, question and answer, brain-storming, scenario, and role play based on predetermined goals. During these sessions, the students were acquainted with concepts like problem and its types, way of finding possible solutions, applying solutions, accepting and solving the problem, brain-storming, and finally making a decision. They also role-played some problems and practiced the stages of problem solving based on the model. The rate of self-handicapping was evaluated in both intervention and control groups using Jones and Rodwalt’s self-administered questionnaire immediately and one month (30 days) after the end of the training sessions. It should be noted that it took each participant 10 min to complete the questionnaire.

### Statistical methods

Considering the research objectives, descriptive statistics including absolute and relative frequency distribution, mean, and standard deviation were used. Independent t-test was used to compare the two study groups. Additionally, paired t-test, chi-square, and repeated measures ANOVA were used to assess the effectiveness of training in each study group.

### Ethical considerations

After getting permission from Shiraz and Jahrom universities of medical sciences and the Ethics Committee of the universities, sampling was done in faculty of nursing, Jahrom University of Medical Sciences. At the beginning of the educational program, the researchers introduced themselves and explained the study objectives and procedures to the students. Then, written informed consents were obtained from the participants. The participants were also assured that all the gathered data would remain confidential.

## Results

The results of analysis of the demographic data revealed that the majority of students in both groups were accommodated in dormitories. Besides, the majority of students’ parents in both groups had diplomas or under diploma degrees. Moreover, most of the students in the two groups had not experienced any crises in their real lives and many of them were single. The qualitative and quantitative demographic characteristics of the participants have been presented in Tables 1 and 2, respectively.

**Table 1:** Comparison of the intervention and control groups regarding qualitative demographic characteristics

Demographic characteristics		Group				*P*-value
		Intervention		Control		
		Number	Percentage	Number	Percentage	
Gender	Female	21	52.5	23	57.5	0.653
	Male	19	47.5	17	42.5	
Residence	Dormitory	26	65.0	25	62.5	0.816
	House	14	35.0	15	37.5	
Marital status	Single	32	80.0	36	90.0	0.210
	Married	8	20.0	4	10.0	
Father’s education level	Illiterate	15	37.5	6	15.0	0.164
	Diploma	14	35.0	22	55.0	
	Associate degree	4	10.0	5	12.5	
	Bachelor’s degree and above	7	17.5	6	17.5	
Mother’s education level	Illiterate	16	40.0	10	25.0	0.237
	Diploma	16	40.0	17	42.5	
	Associate degree	3	7.5	6	15.0	
	Bachelor’s degree and above	5	12.5	7	17.5	
Crisis in life	Yes	12	30.0	10	25.0	0.617
	No	28	70.0	30	75.0	
Date of registration in the university class	2013	12	30.0	12	30.0	0.999
	2014	7	17.5	7	17.5	
	2015	9	22.5	9	22.5	
	2016	12	30.0	12	30.0

**Table 2:** Comparison of the intervention and control groups regarding quantitative demographic characteristics

Quantitative demographic characteristics	Group				*P*-value
	Intervention		Control		
	Mean	Standard deviation	Mean	Standard deviation	
Age	21.35	2.15	21.13	1.77	0.611
Mean of previous semesters’ average points	15.78	0.91	15.64	1.01	0.506

The results of chi-square test revealed no significant differences between the two groups with respect to qualitative and quantitative demographic variables before the intervention (*p* > 0.05). Hence, the two groups were the same regarding qualitative and quantitative demographic characteristics. Furthermore, no significant relationships were observed between demographic characteristics and self-handicapping, ill temper, lack of endeavor, and excusing scores (*p* < 0.05).

The results of repeated measures ANOVA showed that teaching problem solving skills in the intervention group led to a significant decrease in the rate of self-handicapping after the intervention (*p* < 0.001). However, no significant change was observed in the mean score of self-handicapping in the control group (*p* = 0.575). Similar results were also obtained for ill temper, excusing, and lack of endeavor subscales, showing a decrease in the mean scores of the three subscales due to teaching problem solving skills. This indicates the effectiveness of the training.

The results of independent samples t-test revealed no significant difference between the two groups concerning the mean score of self-handicapping immediately after the intervention (*p* = 0.761). However, a significant difference was observed between the two groups in this regard one month after the intervention (*p* = 0.014). This result showed that teaching problem solving skills was effective in the students’ self-handicapping one month after the intervention, but not immediately after that. Moreover, comparison of the two groups’ scores of ill temper, excusing, and lack of endeavor factors only revealed a decline in the mean score of the excusing factor one month after the intervention (Table 3).

**Table 3:** Comparison of the two groups’ mean scores of self-handicapping before and after the intervention

Group	Before the intervention		Immediately after the intervention		One month after the intervention		F	*P*-value
	Mean	Standard deviation	Mean	Standard deviation	Mean	Standard deviation		
Intervention	70.58	11.53	64.73	11.53	59.98	11.70	35.80	0.000
Control	64.70	9.09	65.45	9.66	65.98	9.66	0.320	0.575
T	1.893		−0.305		−2.501			
P-value	0.062		0.761		0.014			

## Discussion

The current study aimed at investigating the effect of teaching problem solving skills on the rate of self-handicapping among nursing students. The results showed that teaching problem solving skills led to a decline in the rate of self-handicapping among the students one month after the intervention.

The results of analyzing the participants’ demographic characteristics showed no significant relationships between the qualitative demographic characteristics and self-handicapping, ill temper, lack of endeavor, and excusing scores. The results also revealed no significant relationships between the participants’ last semester’s average point and lack of endeavor. Up to now, various results have been obtained with regard to self-handicapping due to gender differences. Some studies have shown a difference between males and females concerning the rate of self-handicapping [28, 29], while others have not supported this difference [30]. In the context of academic self-handicapping, Leondari and Gonida (2007) conducted a research on 702 boys and girls from early elementary to late high school grades and reported no significant differences between the two genders with respect to the rate of self-handicapping [31]. This finding can be justified by the fact that challenging situations cause a kind of negative anxiety and stress for all individuals, including both men and women [32]. Since men as well as women need to protect their self-esteem in coping with challenges, both genders try to use strategies such as self-handicapping to retain the community’s view on themselves and their capabilities. Considering the fact that individuals’ thoughts and beliefs change with age, different results obtained in various studies might be attributed to performance of studies among different age groups. For example, adolescence can direct both genders towards using self-handicapping. The contradictory results might also be attributed to performance of studies in different situations and environments, which might be due to the effect of different cultural and social beliefs and perceptions in various circumstances on the formation of both genders’ personality traits.

In the present study, no significant change was observed in the intervention group’s mean score of self-handicapping compared to the control group immediately after the intervention. However, a significant decrease was detected in this regard one month after the intervention. Similarly, Hosseini et al. (2014) demonstrated that cognitive teaching of improving hope caused a significant decrease in the students’ educational self-handicapping after the intervention [33]. In another study conducted by Hosseinian et al. (2011) to investigate the effectiveness of cognitive-behavioral teaching, a significant difference was seen in the intervention group’s mean scores of self-handicapping and self-efficacy in comparison to the control group after the intervention [20]. Various studies have illustrated that individual and group psychological programs played a key role in improving the psychological conditions of service users [34, 35]. According to some researchers, one of the effective factors in humans’ behaviors and feelings is their attitudes and beliefs about bad events that are considered the core of cognitive approaches [36]. Based on the cognitive approach, individuals’ beliefs and cognitive processes are determinants of their feelings and behaviors in different situations [37]. Thus, considering the fact that all people face problems and obstacles in the course of life and the probability of their vulnerability, it is necessary for them to have skills that enable them to deal with their problems in the best way. In a study by Krenz et al. (2008), the cognitive-behavioral intervention played no roles in decreasing the rate of self-handicapping immediately after the training. However, a significant decline was observed in this measure in a proceeding study that was done to check the results one month after the intervention [38]. Since no countercurrent study was found on this subject, according to the researchers’ viewpoints, self-handicapping affected humans’ behaviors despite having a cognitive basis [39].

### Limitations

One of the limitations of this study was that the sample was limited to just one city and it was not possible for the researchers to travel to other cities for sampling. This might affect the generalizability and interpretation of the results.

## Conclusions

The results of this research revealed that the problem solving skills training led to a decline in the rate of self-handicapping in the intervention group compared to the control group. In this research, efforts were made to reinforce effective personal strategies in the students by teaching problem solving skills. Because nursing students experience more stresses compared to the students of other majors, self-handicapping can affect their behaviors including the quality of healthcare they provide for patients in future as well as their psychological health. Therefore, the respected officials and managers of universities are recommended to incorporate problem solving skills training in the students’ curricula or introduce it as a course during their education period.

## Abbreviations

ANOVA: Analysis of variance
NCSS: Numerical cruncher statistical software.
PASS: Power analysis and sample size

## References

[1] Dadsetan P (2001). Development psychology:from children to adult.

[2] Borhani F (2016). Abbas zadeh a, Sabzevari S. efforts for educational justice: explaining the clinical evaluation process of nursing students, a grounded theory study biological. Ethics.

[3] Khademian Z, Pishgar Z, Torabizadeh C. Effect of training on the attitude and knowledge of teamwork among anesthesia and operating room nursing students: a quasi-experimental study. Shiraz E-Med J. 2018; In Press.

[4] Eyink J, Hirt ER, Hendrix KS, Galante E (2017). Circadian variations in claimed self-handicapping: exploring the strategic use of stress as an excuse. J Exp Soc Psychol.

[5] Takeuchi H, Taki Y, Nouchi R, Hashizume H, Sekiguchi A, Kotozaki Y (2013). Anatomical correlates of self-handicapping tendency. Cortex.

[6] Petersen L-E (2014). Self-compassion and self-protection strategies: the impact of self-compassion on the use of self-handicapping and sandbagging. Personal Individ Differ.

[7] Kaya F, Tümkaya S (2017). Investigating the prediction levels of school alienation of the classroom teaching students’ achievement orientation, self handicapping behaviours and demographic features Sınıf öğretmenliği öğrencilerinin başarı yönelimi, kendini engelleme davranışları ve demografik özelliklerinin okula yabancılaşmayı yordama düzeylerinin incelenmesi. J Human Sci.

[8] Berglas S, Jones EE (1978). Drug choice as a self-handicapping strategy in response to noncontingent success. J Pers Soc Psychol.

[9] Finez L, Sherman DK (2012). Train in vain: the role of the self in claimed self-handicapping strategies. J Sport Exerc Psychol.

[10] Antony J (2016). Academic self-efficacy and self-handicapping: are they influenced by self-regulated learning?. J Res The Bede Athenaeum.

[11] Uysal A, Knee CR (2012). Low trait self-control predicts self-handicapping. J Pers.

[12] Karner-Huţuleac A (2014). Perfectionism and self-handicapping in adult education. Procedia Soc Behav Sci.

[13] Clarke IE, MacCann C (2016). Internal and external aspects of self-handicapping reflect the distinction between motivations and behaviours: evidence from the self-handicapping scale. Personal Individ Differ.

[14] Schwinger M (2013). Structure of academic self-handicapping—global or domain-specific construct?. Learn Individ Differ.

[15] Gadbois SA, Sturgeon RD (2011). Academic self-handicapping: relationships with learning specific and general self-perceptions and academic performance over time. Br J Educ Psychol.

[16] Snyder KE, Malin JL, Dent AL, Linnenbrink-Garcia L (2014). The message matters: the role of implicit beliefs about giftedness and failure experiences in academic self-handicapping. J Educ Psychol.

[17] Zabihollah K, Yazdani Varzaneh MJ, Gholamali Lavasani M (2012). Self-efficacy and self-handicapping in high school students. Transform Psychol (Iranian Psychologists).

[18] Molaie A, Shahidi S, Vazifeh S, Bagherian S (2010). Comparing the effectiveness of cognitive behavioral therapy and movie therapy on improving abstinence self-efficacy in Iranian substance dependent adolescents. Procedia Soc Behav Sci.

[19] Bikmohamadi M, Tarkhan M, Akbari B (2013). The effect of group cognitive-behavioral training and coping with stress on social adjustment and self-concept of high school female students. Knowledge Res Appl Psychol.

[20] Hosseinyan S, Niknam M (2011). The effect of cognetive behavior thearapy on self handicapping and self efficacy on woman athletes journal of behavioral and sports. Psychology.

[21] Jafarzadeh Ghadimi A, Emampour S, Sepah Mansour M, Jafarzadeh Ghadimi M (2013). Comparison of problem-solving styles and self-efficacy beliefs in high school students in typical and normal schools education and evaluation (educational). Sciences.

[22] Kimiaei SA, Karimi F, editors. Problem solving training and its effect on reducing conflicts and improving emotional literacy of teenage girls with addicted parents. Kuala Lumpur: The IRES-international conference of education and social sciences (ICESS); 2015.

[23] Malouff JM, Thorsteinsson EB, Schutte NS (2007). The efficacy of problem solving therapy in reducing mental and physical health problems: a meta-analysis. Clin Psychol Rev.

[24] Hwang G-J, Hung C-M, Chen N-S (2014). Improving learning achievements, motivations and problem-solving skills through a peer assessment-based game development approach. Educ Technol Res Dev.

[25] Heidari M, Shahbazi S. The impact of training by social problem-solving model of D–zurilla & gold fried on problem-solving skills of nursing students. Iran J Nurs. 2012;25(76):1–9.

[26] Shamsikhani S, Farmahini Farahani M, Shamsikhani S, Sobhani M (2014). Effectiveness of problem solving training on depression in nursing student. Int J Palliat Nurs.

[27] Heidari M, Khodapanahi M, Dehghani M (2009). Psychometric examination of self-handicapping scale (SHS).

[28] Brown CM, Park SW, Folger SF (2012). Growth motivation as a moderator of behavioral self-handicapping in women. J Soc Psychol.

[29] Midgley C, Urdan T (1995). Predictors of middle school students' use of self-handicapping strategies. J Early Adolesc.

[30] Feick DL, Rhodewalt F (1997). The double-edged sword of self-handicapping: discounting, augmentation, and the protection and enhancement of self-esteem. Motiv Emot.

[31] Leondari A, Gonida E (2007). Predicting academic self-handicapping in different age groups: the role of personal achievement goals and social goals. Br J Educ Psychol.

[32] Vakilian S, Ghnbari H (2009). Effect of cognitive-behavioral group therapy in combination with social skill training on fear of negative evaluation and social avoidance. J Clin Psychol.

[33] Hosseni A, Salimi H (2014). Esa zadegan a. the effect of cognitive hope enhancing training on reduction of academic procrastination and self-handicapping in boy students of boarding guidance schools in Boukan. J Edu psychol Stud.

[34] Dobson D, Dobson KS. Evidence-based practice of cognitive-behavioral therapy. New York London: Guilford publications; 2018.

[35] Ehlers A, Bisson J, Clark DM, Creamer M, Pilling S, Richards D (2010). Do all psychological treatments really work the same in posttraumatic stress disorder?. Clin Psychol Rev.

[36] Ciarrochi J, Bailey A. A CBT-practitioner's guide to ACT: how to bridge the gap between cognitive behavioral therapy and acceptance and commitment therapy. Oakland: New Harbinger Publications; 2008.

[37] Weiner DA, Schneider A, Lyons JS (2009). Evidence-based treatments for trauma among culturally diverse foster care youth: treatment retention and outcomes. Child Youth Serv Rev.

[38] Kearns H, Forbes A, Gardiner M (2007). A cognitive behavioural coaching intervention for the treatment of perfectionism and self-handicapping in a nonclinical population. Behav Chang.

[39] Robert L (2008). Cognitive therapy technique.

